# Optimizing Non-Thermal Magnetic Field to Minimize Weight Loss and Tissue Degradation: Identifying Possible Enzyme Inhibition Mechanisms

**DOI:** 10.3390/foods14020166

**Published:** 2025-01-08

**Authors:** Chao-Kai Chang, Prakoso Adi, Rizka Mulyani, Chun-Fu Lin, Ratna Sari Listyaningrum, Shella Permatasari Santoso, Mohsen Gavahian, Chang-Wei Hsieh

**Affiliations:** 1 Department of Food Science and Biotechnology, National Chung Hsing University, Taichung City 402202, Taiwan; 2 International Doctoral Program in Agriculture, National Chung Hsing University, Taichung City 402202, Taiwan; 3 Department of Agricultural Product Technology, Sebelas Maret University, Surakarta City 57126, Indonesia; 4 Department of Medicinal Botanicals and Health Applications, Da-Yeh University, Chang-Hua 515006, Taiwan; 5 Department of Food Technology, Muhammadiyah University of Bandung, Bandung City 40614, Indonesia; 6 Department of Chemical Engineering, Widya Mandala Surabaya Catholic University, Surabaya 60114, Indonesia; 7 Department of Chemical Engineering, National Taiwan University of Science and Techology, Taipei 106335, Taiwan; 8 Department of Food Science, National Pingtung University of Science and Technology, Pingtung 912301, Taiwan; 9 Department of Food Science, National Ilan University, Yilan City 260007, Taiwan; 10 Department of Medical Research, China Medical University Hospital, Taichung City 404327, Taiwan

**Keywords:** emerging technologies, magnetic field, orthogonal experiment, sustainable preservation, process optimization, tissue degrading enzyme

## Abstract

This research investigates potential mechanisms of novel magnetic field (MF) treatments in inhibiting cell-wall-degrading enzymes, aiming to reduce weight loss and preserve the post-harvest quality of tomatoes (*Solanum lycopersicum* L.) as a climacteric fruit. The optimization of the processing parameters, including MF intensity (1, 2, 3 mT), frequency (0, 50, 100 Hz), and duration (10, 20, 30 min), was accomplished by applying an orthogonal array design. In particular, the investigation delved into the underlying mechanisms by which MF impedes the activity of tissue-degrading enzymes, such as pectin esterase (PE), polygalacturonase (PG), and cellulase (Cx), during the storage period. The results showed that MF treatment delayed the increase in soluble solids by 1.5 times and reduced titratable acidity by 1.2 times. The optimal treatment conditions—2 mT, 50 Hz, and 10 min—achieved the most significant inhibition of weight loss (4.22%) and maintained tissue integrity for up to 21 days. Optimized MF significantly suppressed enzyme activity, with PE activity reduced by 1.5 times, PG by 2.8 times, and Cx by 2.5 times. Also, cross-sectional images and external appearance demonstrated that MF-treated tomatoes retained their internal tissue structure throughout the extended storage period. These findings suggest that MF treatments can effectively suppress the key enzymes responsible for tissue degradation, ultimately delaying weight loss and softening, preserving post-harvest quality, and contributing to sustainable food production and zero waste.

## 1. Introduction

As global living standards improve and agricultural technologies advance, consumers are increasingly demanding fresher fruits and vegetables while also striving to minimize food waste. Unlike fresh meat and other agricultural products, climacteric fruits remain living organisms after harvest. Fresh climacteric fruits undergo various physiological processes after harvest, such as respiration and transpiration, which lead to weight loss and reduce or diminish their commercial value [1,2]. Climacteric fruits, such as tomatoes, are particularly susceptible to damage during storage, often resulting in significant waste. This vulnerability stems from the post-harvest increase in respiration, which leads to the production of large amounts of ethylene [3]. Ethylene is crucial for the activation of tissue-degrading enzymes, such as pectin esterase (PE), polygalacturonase (PG), and cellulase (Cx), which are responsible for fruit ripening [4]. The enzymatic activity accelerates fruit softening and weight loss [5], culminating in fruit decay. This underscores the critical need for innovative preservation techniques.

Traditional preservation strategies encompass chemical preservation, which involves the application of ethylene inhibitors to reduce ethylene biosynthesis during storage [4]. Modified atmosphere preservation (MAP) is another widely used method, utilizing low-temperature storage with precisely controlled oxygen and carbon dioxide (CO_2_) levels to slow respiration, inhibit ethylene production, and retain nutrient quality [6]. However, these methods have inherent limitations, including the potential for quality degradation over time, concerns about chemical residues, and the high costs associated with equipment and specialized labor.

The demand for safer, sustainable methodologies that do not pose health risks steadily increases, and emerging food processing technologies, such as electrical-based technologies, have been proposed to achieve this [7]. High-voltage electrostatic fields (HVEFs), for example, generate electric currents that alter cell membrane potentials, affecting the movement of charged particles and driving biochemical reactions. Studies have demonstrated that using a 600 kV/m electric field for two hours can reduce weight loss in tomatoes by up to six times [8]. HVEFs have been successfully applied to extend the shelf life of fish and fruit juice while reducing bacterial loads during cold storage [9,10]. Despite these promising outcomes, this technology has challenges. The potential for irreversible damage to cell membranes may adversely affect fruit texture and marketability, especially for delicate produce [11]. Magnetic fields (MF) are another option with advantages such as a minimal dependence on the size and shape of the product, less energy consumption, and highly industrial adaptability [12]. Compared with HVEFs, MFs do not generate excessive heat, thereby maintaining food’s structural integrity [13], nutritional content [14], and sensory qualities [12] more effectively. The strong penetrability of MF further enhances its capability to extend the shelf life and maintain the quality of fruits and vegetables [15]. Moreover, MFs redistribute charged ions across cell membranes through the Lorentz force, which alters cellular metabolism in fruit. This process potentially modulates ion distribution in biological systems [16], reducing water loss during storage [17].

Previous research has shown the ability of MFs to improve agricultural product storage quality [15]. However, investigating the specific applications of MFs in fruit post-harvest physiology remains limited [15]. Therefore, this study examines the novel potential mechanism of MF treatments in inhibiting cell-wall-degrading enzymes (PE, PG, Cx) to reduce weight loss and preserve the quality of post-harvest tomatoes. This study also optimized the optimal processing parameters of the MF using an orthogonal array design, including field strength, frequency, and treatment duration.

## 2. Materials and Methods

### 2.1. Sample Preparation

The fresh tomatoes (*Solanum lycopersicum* L. cv. ChenFu 994), a commercial cultivar with a long shelf life, were supplied by Qiao-Yi-Deng Farm Co., Ltd. (Changhua, Taiwan), and seeds were acquired from All Lucky Seed Co., Ltd. (Taipei, Taiwan). Each experiment used ten tomatoes with an average weight of 130 ± 5 g. The tomatoes used in this study were freshly harvested on the same day before reaching the peak respiration phase without visible damage or discoloration, ensuring the uniformity of the samples.

### 2.2. Preliminary Experiments and Selection of Optimal Conditions of the MF Using an Orthogonal Array Design (OAD)

**Figure 1:**
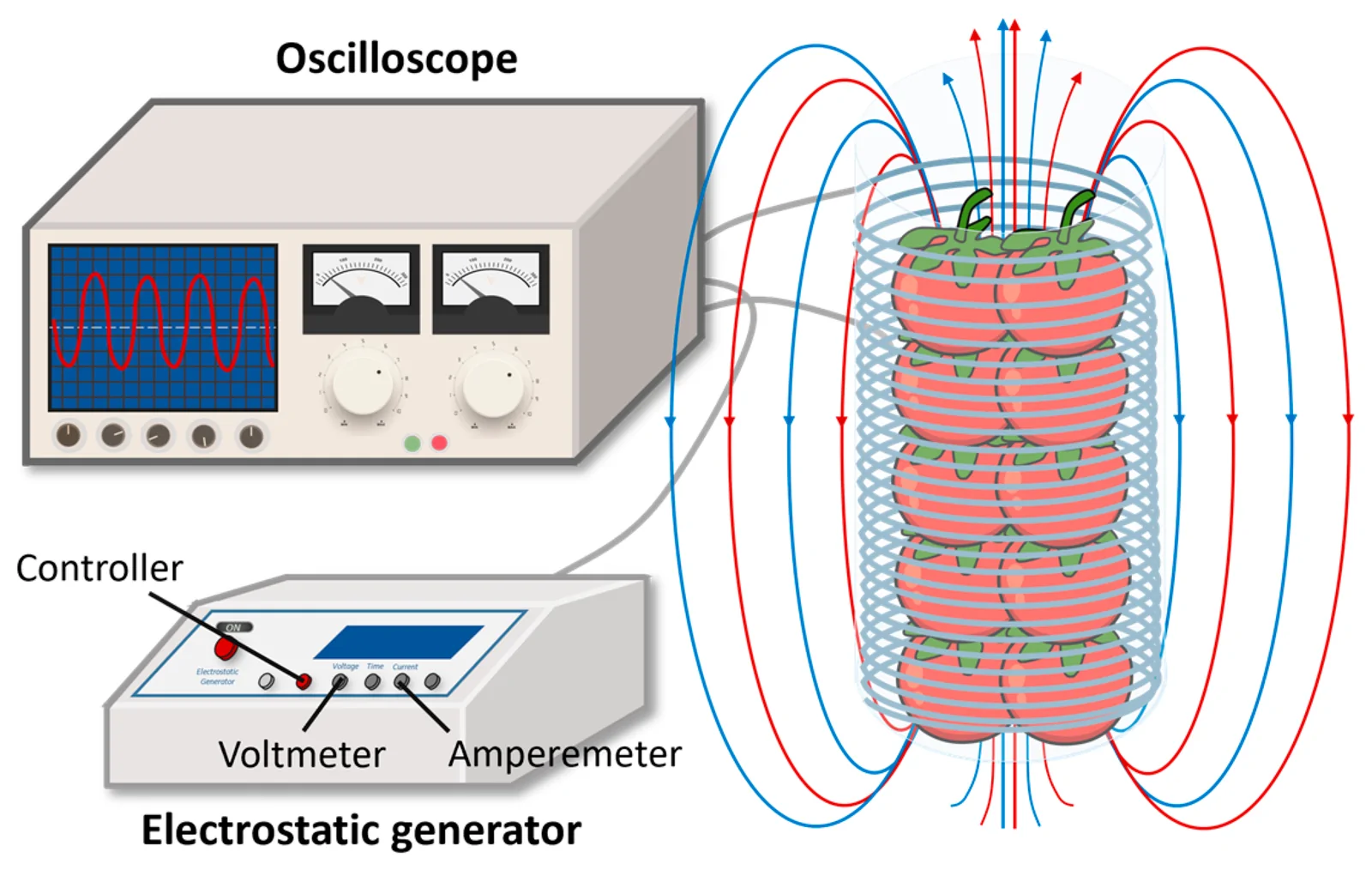
Schematic representation of the MF treatment system.

The magnetic field used in this study followed the previous research [18,19]. A customized electromagnetic generation system (AC power supply, input voltage: 120 V; output voltage: 20 V; adjustable frequency: 0 Hz, 50 Hz, and 100 Hz; adjustable magnetic field intensity: 0.94–3.11 mT) was provided by He-Kang Electromagnetic Technology Co., Ltd., Hsinchu, Taiwan. This system was connected to the Helmholtz coil to generate an MF effect with bipolar wave pulses. Tomato samples were placed within an MF for processing, and the electromagnetic parameters were continuously monitored using an oscilloscope (TBS 1052C, Tektronix, Inc., Beaverton, OR, USA) throughout the process (Figure 1).

An orthogonal experimental design was used, with different MF intensities of 1, 2, and 3 mT and different MF frequencies of 0, 50, and 100 Hz applied to the tomatoes for 10, 20, and 30 min. The experimental conditions during the MF treatment were maintained at 25 °C using an air-cooling machine (RAC-4AQ7, Hitachi, Ibaraki, Japan) with an air velocity that did not exceed 30 m^3^/min and at an RH of 87.5 ± 2.5% using a dehumidifier (AD-BH333FT, Sampo, Taoyuan, Taiwan). The tomatoes were stored at room temperature for 15 days for the preliminary study. The sample weight loss rate was measured to understand the effects of different conditions on the physical changes in the tomatoes during storage. Subsequently, the tomatoes were stored for 21 days at room temperature under the optimized MF conditions, and experiments on the quality indicators were conducted. The room temperature storage conditions were maintained at 25 °C with an air velocity not exceeding 30 m^3^/min and an RH of 87.5 ± 2.5%.

A preliminary test was conducted to evaluate the optimal MF conditions for treating tomatoes, and an experimental range was selected. This study utilized an L9 OAD for the initial experiments on weight loss rate, employing MF strength, frequency, and treatment time as the experimental factors (Table 1) using Minitab^®^ v22.1. Each factor had three levels, including MF strength (1 mT, 2 mT, 3 mT), frequency (0 Hz, 50 Hz, 100 Hz), and treatment time (10, 20, and 30 min), as detailed in Table 1. The experiment was conducted with 9 groups of experimental configurations, and the results were analyzed to deduce the optimal combination of experimental factors and levels. Ten tomato fruits were used for each test and repeated thrice.

### 2.3. Quality Index

#### 2.3.1. Appearance and Weight Loss Rate

Whole tomatoes and cross-sectional cuts were photographed to observe the visual morphological changes in the tomatoes at the beginning and end of storage. The weight of the tomatoes was measured after harvest to record their pre-treatment weight. After undergoing different treatment conditions, the tomatoes were stored at room temperature. A fixed electronic balance (MR204, Mettler-Toledo Ltd., Leichester, UK) was used for the weight measurements, and the weight loss rate was calculated using Equation (1); the measurements were repeated 10 times [20]:

(1) $$W\;\left(\%\right)=\frac{\left(B-K\right)}{B}\times 100\%$$

where A is the weight loss (%), B is the weight before storage (g), and K is the weight after storage (g).

#### 2.3.2. Soluble Solids

One gram (1 g) of tomato pulp was mixed with deionized water to prepare a 1% tomato juice solution. The soluble solid content was then measured using a refractometer (Master 500, Atago Co., Ltd., Tokyo, Japan) at 25 ± 0.5 °C, with the result expressed in Brix units.

### 2.4. Titratable Acidity

Fresh tomatoes were ground in a mixer (Oster 6640, Sunbeam Products, Inc., Boca Raton, FL, USA) and filtered using a coarse filter (Uni-Laiyie, Lai Yi Industrial Co., Ltd., Tainan, Taiwan). The 5 mL filtrate was then collected and supplemented with two drops of phenolphthalein (Katayama Chemical Co., Ltd., Osaka, Japan) indicator. Subsequently, the solution was titrated with 0.1 N NaOH (Katayama Chemical Co., Ltd., Osaka, Japan) until a color change occurred. The titration volume was recorded and substituted into the formula. The percentage of acidity was calculated as citric acid (Katayama Chemical Co., Ltd., Osaka, Japan) per 100 g of tomato using the following equation [21]:

(2) $$Acidity\%=\frac{Vol.NaOH\left(mL\right)\times 0.1\;(\mathrm{Normality}\;\mathrm{of}\;\mathrm{NaOH})\times 0.064}{mL\;tomato\;juice}\times 100$$

where 0.064 is the citric acid milliequivalent factor.

### 2.5. Tissue Degradation Enzyme Activity During Storage

#### 2.5.1. PE Activity

Enzyme extraction was performed following a previously employed method [22] with slight modifications. After peeling the tomatoes, the flesh was mixed with an extraction buffer containing 2% NaCl solution (*w*/*v*) (Katayama Chemical Co., Ltd., Osaka, Japan) at 4 times the sample’s initial weight. The homogenization process was conducted for 2 min using a blender (Oster 6640, Sunbeam Products, Inc., Boca Raton, FL, USA), followed by centrifugation at 10,000× *g* for 50 min at 4 °C. The supernatant was collected as a crude PE extract. The PE activity was measured according to the procedure described by [23]. In brief, 15 mL of a 0.5% citrus pectin solution containing 0.1 M NaCl (Katayama Chemical Co., Ltd., Osaka, Japan) was adjusted to pH 6.5. Subsequently, 1 mL of the crude PE enzyme solution was added to the mixture. The carboxyl groups produced during the reaction were titrated with a 0.01 M NaOH solution (Katayama Chemical Co., Ltd., Osaka, Japan). The reaction time was recorded, and one unit of PE activity was defined as the amount of enzyme required to produce one equivalent of carboxyl per minute. The PE activity (unit) is calculated using the formula following [22], as shown in Equation (3):

(3) $$PE\;activity\;(unit)\;=\frac{\left(A\times F\times 0.01\right)}{\left(T\times 1000\times W\right)}$$

where A is the volume of 0.01 M sodium hydroxide (PubChem CID: 14798) solution (mL), F is a factor of 0.01 M sodium hydroxide solution, T is a reaction or titration time (min), and W is the dilution factor.

#### 2.5.2. PG Activity

Enzyme extraction was performed following a method used in a previous study [22] with slight modifications. Following the peeling of the tomatoes, the flesh was mixed with Tris buffer (pH 7.0, *w*/*v*) (Omics Bio, Taipei, Taiwan) at 10 times the sample’s initial weight. The mixture was then homogenized (DSR-2100P, Digisystem Laboratory Instruments, New Taipei, Taiwan) for 2 min and centrifuged (Himac CT 18R, Koki Holdings Co., Ltd., Tokyo, Japan) at 9894.3× *g* for 50 min. The supernatant was collected to obtain the crude enzyme for analyzing PG and Cx activity. All procedures were carried out at 4 °C.

The activity of PG was determined according to the method described by Chang et al. [22] with slight modification. Briefly, 0.4 mL of the crude PG enzyme solution was added to 1% polygalacturonic acid in 200 mM acetate buffer (pH 4.5). The reaction mixture was incubated in a water bath at 37 °C for 1 h. Following the incubation, 1 mL of 3,5-dinitro salicylic acid (DNS) reagent (Sigma-Aldric, Darmstadt, Germany) was added, and the mixture was heated in a boiling water bath (SB-1200, EYELA, Bohemia, NY, USA) for 5 min to inactivate the enzyme and terminate the reaction. The absorbance of the resulting solution was measured at 550 nm. For the blank test, the enzyme was pre-inactivated by heating before the reaction. A standard curve was constructed using galacturonic acid solutions ranging from 0 to 5000 μg/mL. Polygalacturonase (PG) activity was defined according to Chang, Tsai et al. [22], where 1 unit (U) of enzyme activity is the amount of enzyme required to release 1 μmol of galacturonic acid from polygalacturonic acid per minute.

#### 2.5.3. Cx Activity

The procedure for Cx enzyme extraction was in line with the PG enzyme extraction. An amount of 0.5 mL of the crude Cx enzyme solution was added to a 1% carboxymethyl cellulose (CMC) solution (Sigma-Aldric, Darmstadt, Germany). The reaction mixture was incubated in a water bath (SB-1200, EYELA, Bohemia, NY, USA) at 37 °C for 1 h. Following incubation, 1 mL of DNS reagent (Sigma-Aldric, Darmstadt, Germany) was added, and the mixture was heated in a boiling water bath for 5 min to inactivate the enzymes and terminate the reaction. The absorbance of the reaction mixture was measured at 550 nm. For the blank test, the enzyme was pre-inactivated by heating before the reaction. A standard curve was constructed using glucose solutions ranging from 0 to 3600 μg/mL. One unit of enzyme activity was defined as the amount of enzyme required to catalyze the formation of 1 μmol of the reducing groups per minute per gram of the original fresh flesh weight, expressed as U mg^−1^, using glucose as the standard [24].

### 2.6. Enzyme Kinetics

An enzyme kinetic analysis to determine K_m_ and V_max_ was conducted following [25] with slight modification. Commercial citrus pectin (methoxy content: 9.5%) was prepared at varying concentrations (0.2–1%) as the substrate for the enzyme reactions for PE and PG. Meanwhile, 1% CMC solution (Sigma-Aldric, Darmstadt, Germany) was prepared at varying concentrations (0.25–3%) as the substrate for the Cx enzyme reaction.

The reaction rates of tomato PE, PG, and Cx enzymes were plotted against the substrate concentration using a double reciprocal plot (Lineweaver–Burk), allowing for the determination of K_m_ and V_max_ values. Based on the Lineweaver–Burk graph, two critical terms in enzyme kinetics could be determined: K_m_ (Michaelis constant) and V_max_ (maximum velocity). The y-intercept of such a graph corresponds to the inverse of Vmax, while the x-axis intersection represents −1/K_m_. V_max_ and K_m_ could, therefore, be determined experimentally or calculated from the Lineweaver–Burk equation [26]. Variations in K_m_ and V_max_ were observed, and relative enzyme activity, along with Lineweaver–Burk plots (1/V versus 1/s), were utilized to characterize the type of enzyme inhibition (competitive, noncompetitive, and uncompetitive).

### 2.7. Statistical Analysis

The OAD results and data processing were statistically interpreted with Minitab^®^ v22.1 (v22.1, Minitab, LLC., State College, PA, USA). Statistical significance was assessed at *p* < 0.05 with a one-way analysis of variance (ANOVA) using IBM SPSS 22 software (v22, IBM Corp., Armonk, NY, USA). Differences in the means between the optimal MF effect and the control for tomatoes were determined by an unpaired *t*-test (*p* < 0.05) for comparative analysis. Ten replicates were used to calculate the mean ± standard deviation (SD) for the preliminary experiments, the selection of optimal MF conditions, the assessment of quality attributes during storage, including weight loss, soluble solids, and titratable acidity, and the analysis of tissue-degrading enzyme activities; whereas three replicates were used to calculate the mean ± SD for the analysis of the enzyme inhibition.

## 3. Results and Discussion

### 3.1. Optimization of the MF Treatment Using OAD

The present study evaluated the effect of MF treatment on weight loss in fresh tomatoes stored under ambient conditions, simulating typical market environments. The weight loss of fruit is a significant indicator in storage and preservation, as it is directly related to quality attributes, including firmness and visual appearance [27]. Therefore, we evaluated the weight loss in this first step to find the optimized condition for the MF treatment. The key MF parameters—intensity, frequency, and processing time—were optimized using an OAD to minimize tomato preservation weight loss over a 15-day storage period at 25 °C.

**Figure 2:**
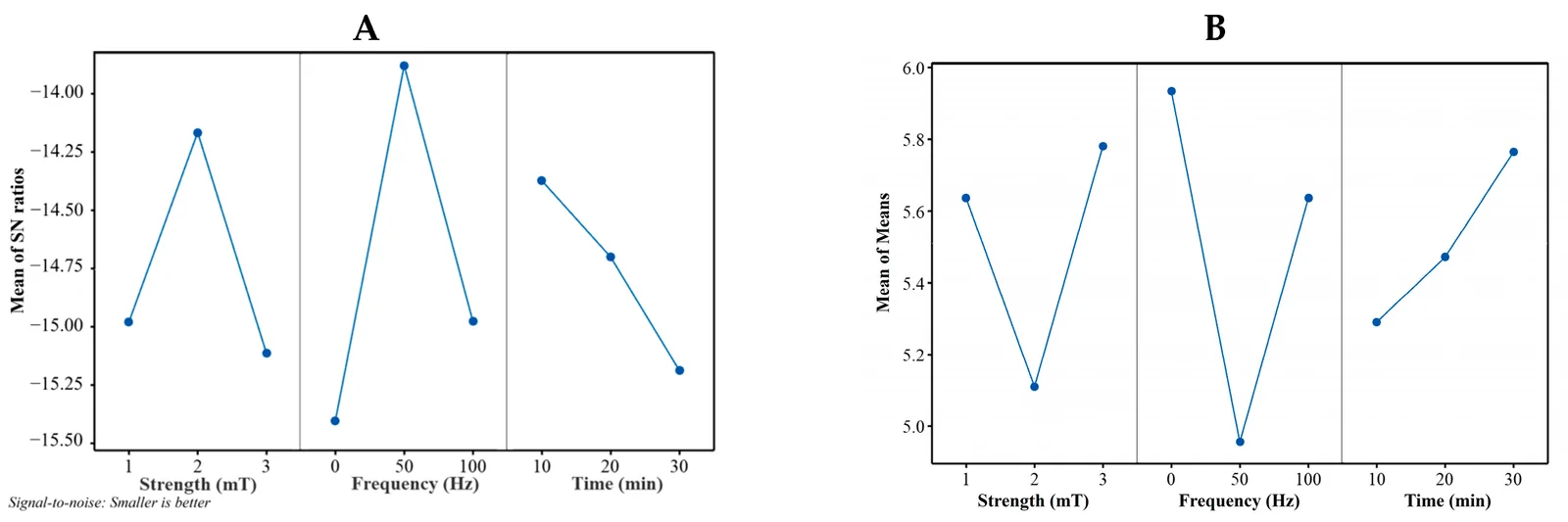
Analysis of the orthogonal array design (OAD) for (**A**) signal-to-noise (SN) ratio and (**B**) means of the impact of the optimization factors on the weight loss of tomatoes.

Table 1 shows the OAD and analysis of the weight loss of tomatoes. The treatment conditions of sample 8 (3 mT, 50 Hz, and 10 min) resulted in lower tomato weight loss rates, with values of 4.52%. In comparison, a higher weight loss in tomatoes was observed in sample 9 (3 mT, 100 Hz, and 20 min). After calculating the means of the percentage weight loss derived from the respective factor levels, R (delta) was calculated by subtracting the highest and lowest k1, k2, and k3, indicating the factors’ contribution to the treatment outcome. According to the factor contribution, frequency has the highest impact, followed by the strength and time of the MF treatment, on preserving tomatoes. To identify the optimal combination of the process parameters, the signal-to-noise ratio (S/N) was calculated to reduce the noise factors. Since this study is aimed at a lower weight loss, the S/N ratio targets to achieve that ‘the smaller is better’ were used (Figure 2A).

Our results highlighted the different MF frequencies (0, 50, and 100 Hz) that showed significantly different values of the percentage of weight loss (*p* < 0.05), as can be seen in Table 2. Notably, the OAD analysis in Figure 2B showed that applying an MF frequency of 50 Hz to tomatoes resulted in a 5% weight loss. Meanwhile, during storage, 0 Hz and 100 Hz led to higher weight losses of 5.88% and 5.67%, respectively. This finding aligns with a previous study that showed that under similar MF settings (50 Hz, 1–4 mT), bananas displayed the lowest weight loss (10%) compared to other treatments after 15 days of storage at 4 °C [28]. Similarly, applying 50 Hz at a magnetic strength of 1–5 mT in strawberries reduced weight loss by up to 25%, outperforming both the 0 Hz and 100 Hz treatments [29]. These previous studies demonstrate that under similar MF conditions, the weight loss of tomatoes stored at 25 °C surpassed those stored at a cooled temperature of 4 °C. However, increasing the frequency over 50 Hz can cause microbursts in fruit cells, leading to excessive water release and significantly higher weight loss [30]. Gąstoł et al. [31] identified that an MF primarily affects plant tissue via structural integrity water content during treatment. An increase in the frequency of an MF is hypothesized to enhance enzymatic activity in plant tissue, leading to cell wall degradation [31]. This degradation is expected to reduce water-holding capacity (WHC), resulting in increased water loss in fruit [22]. Therefore, the optimal magnetic frequency for this experiment is 50 Hz.

According to the variance analysis result (Table 2), the effect of MF strength and time on weight loss did not significantly affect weight loss during tomato storage at 25 °C. The OAD analysis confirmed that the optimization of strength and time are ranked second and third in affecting the MF process, respectively (Table 1). According to Figure 2B, when intensity was increased from 1 mT to 2 mT, the response value showed a downward trend, and the weight loss rate dropped from 5.68% to 5.07%, but when 2 mT was increased to 3 mT, the weight loss rate increased from 5.07% to 5.78%. In addition, the result showed that the weight loss of tomatoes during storage at 10, 20, and 30 min was 5.45%, 5.31%, and 5.77%, respectively (Figure 2B). These data indicate that the greater the time applied, the greater the weight loss in tomatoes during storage. Other studies have shown that treating a low MF (less than 3 mT) needs more time to pronounce the preserving effect on an agricultural product. For instance, a low MF requires 8 h of treatment to achieve an 8% weight loss for a more extended storage period of 21 more days [11]. Our study demonstrated that a shorter duration of MF treatment (10 min) can reduce weight loss by twofold compared to a previous study [11].

MFs have been investigated for their capacity to change the physical and chemical processes in fruits, potentially affecting weight loss during storage [19]. This phenomenon occurs because exposure to the MF causes the water molecules in fruit to become more ordered due to the influence of the Lorentz force, which restricts their movement. As a result, water evaporation from the fruit during storage is inhibited by applying the MF, reducing weight loss [32]. A previous study by Liu et al. [15] demonstrated that 4 mT of MF treatment could inhibit strawberries’ water evaporation (water loss). The authors deduced that an MF could restrict water molecule movement as affected by the Lorentz force from the magnetic field. Based on these findings, we speculate that exposure to a 2 mT MF in our study may similarly induce an ordering effect on water molecules in tomatoes due to the influence of the Lorentz force, thereby restricting their movement.

The MF treatment for preserving tomatoes at 25 °C demonstrated a twofold lower weight loss than a similar MF treatment (4 mT, 30 min, 50 Hz) for 15 days to preserve strawberries in a refrigerator at 4 °C [15]. In addition, the MF treatment in the current study showed a lower weight loss than other non-thermal treatments (HVEF) on cherry tomatoes during storage at 15 °C. On the 12th day, the HVEF weight loss reached 7%, while the MF only reached 5.07% on the 15th day [33]. A previous study proposed that an MF can affect the water dynamics in tomatoes by preserving membrane integrity, which is crucial for minimizing weight loss during storage [34]. The effects of MF strength, frequency, and treatment time on weight loss were analyzed in this study, and the optimal conditions were identified. The results of these predictions were the values calculated by the OAD after applying optimal conditions to the MF configuration for minimal weight loss. Therefore, the optimal weight loss predicted by the OAD analysis was 4.35% with 2 mT, 50 Hz, and 10 min, while the experimental confirmation gave a result of 4.52%. This value was still within the 95% confidence interval predicted by the OAD (4.1325–4.5675%). Previous studies have suggested that the prediction result must have a value very close to the experimental result (observed parameter) [35]. Therefore, the MF conditions of 2 mT, 50 Hz, and 10 min will be used for subsequent analysis.

### 3.2. Effect of MF Treatment on Tomato Quality During Storage

#### 3.2.1. Tomato Appearance

After the fruit is harvested, there is an increase in respiration, which leads to a rise in the rate of ethylene biosynthesis. This condition increases ethylene biosynthesis and enhances enzymatic activity responsible for tissue breakdown. These enzymes promote the breakdown of the cell wall, causing the fruit to lose its structural integrity. This phenomenon accelerates fruit spoilage, promoting pathogen growth, respiration, and ethylene production, causing substantial water loss. Therefore, the fruit’s appearance and cross-sectional images are essential for assessing its quality.

**Figure 3:**
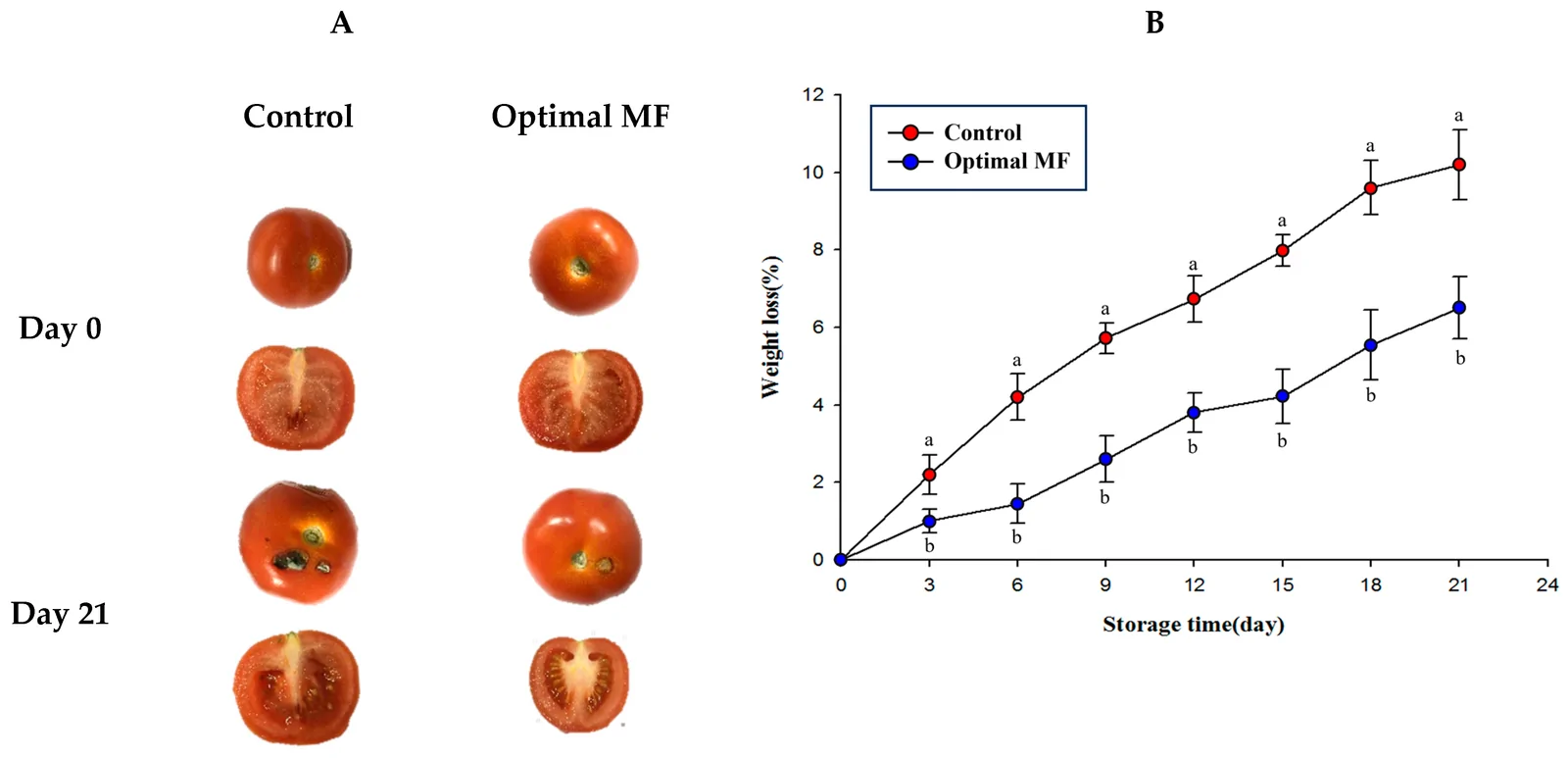

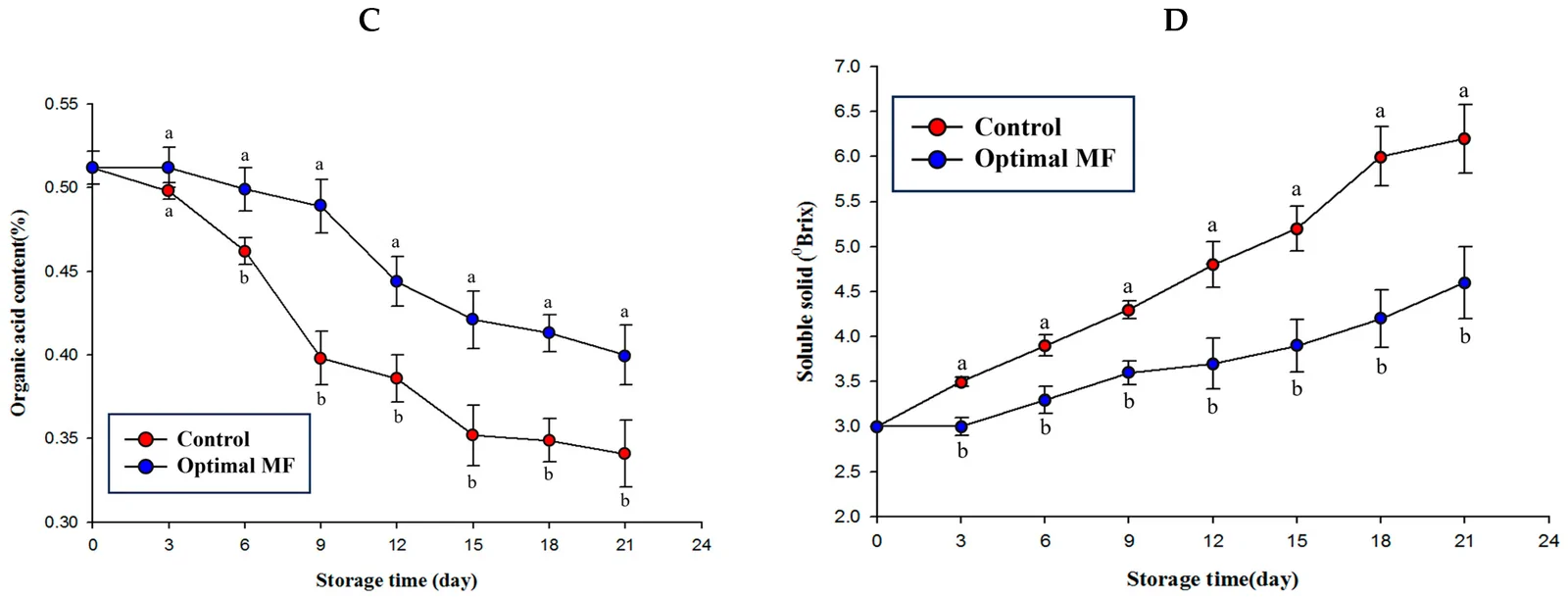
Effect of optimal MF parameters on the (**A**) appearance, (**B**) weight loss (%), (**C**) organic acid content (%), and (**D**) soluble solid content (°Brix) of tomatoes during storage for 21 days at 25 °C (*n* = 10). The MF group was treated with an MF strength of 2 mT, a frequency of 50 Hz, and a treatment time of 10 min; the untreated control group was stored in an environment at 25 °C for 21 days. Letters a and b indicate statistical differences (*p* < 0.05) using unpaired *t*-test.

After 21 days of storage, untreated tomatoes exhibited noticeable wrinkling, mold growth, and indentations in their appearance (Figure 3A). The cross-sectional images also revealed severe autolysis of the tissue structure. However, the MF-treated tomatoes showed a delayed onset of these effects. After being harvested, fruits are categorized into climacteric and non-climacteric based on respiration and ethylene biosynthesis, with significant differences in their ripening processes [36]. Unlike fresh meat and other agricultural products, climacteric fruits remain living organisms after harvest. Fresh climacteric fruits undergo various physiological processes after harvest, such as respiration and transpiration, which lead to weight loss and reduce or diminish their commercial value [1,2]. Climacteric fruits like tomatoes typically experience higher water loss rates than non-climacteric fruits under similar conditions, with tomatoes losing 0.24% water per day at 10 °C storage compared to 0.04% per day for grapefruits [37].

#### 3.2.2. Weight Loss

According to Figure 3B, the weight loss rate of untreated tomatoes after 21 days of storage was 10.02%, while those treated with an MF showed a delayed reduction in weight, with a weight loss rate of 6.01%. This suggests that MF treatment can reduce weight loss in tomatoes during storage. The results also show that the weight loss rate of the MF group was 1.6 times lower than that of the control group. A previous study found that the weight loss of green bell peppers gradually increased with increased storage time [38]. It was discovered that reducing weight loss was more effective when water content was better preserved. Exposure to an MF enhances the structural characteristics of the fruit and reduces water loss from its tissues [11]. MF treatment with a strength of 4 mT and a frequency of 50 Hz significantly improved post-harvest quality, resulting in fewer defects, reduced weight loss, and better firmness retention [15].

#### 3.2.3. Organic Acid

Once detached from the plant, climacteric fruits produce energy to sustain life by consuming organic substances such as carbohydrates, proteins, and acids. As the ripening process progresses, respiration and metabolic activities cause a gradual decline in organic acid content [39]. According to Figure 3C, the untreated tomatoes had an organic acid content of 0.34% after 21 days of storage, while the MF-treated tomatoes had a slower loss of organic acids, with a content of 0.41%. This indicates that MF treatment can preserve the structural integrity of tomato tissues, delay the absorption and conversion of organic acids into energy during storage, reduce the consumption of organic substances, and extend shelf life. Although a previous study demonstrated minor changes in the titratable acidity [19], the difference between the MF-treated and control groups in this study was 1.2 times.

#### 3.2.4. Soluble Solid

Climacteric fruits undergo respiration after being harvested to stay alive, which continues as they ripen. As respiration intensifies, the fruit’s tissues break down, deteriorating the pulp and cell walls. Enzymes that break down tissue cause pectin and cellulose to degrade, producing soluble solids containing soluble sugars [40]. These sugars are the reason for the increased sweetness of the fruit. According to Figure 3D, the untreated tomatoes had a soluble solid content of 6.22 °Brix after 21 days of storage.

In contrast, tomatoes treated with an MF exhibited a delayed release of soluble solids, from 3.0 to 4.4 °Brix. A rapid increase in soluble solid content in climacteric fruits indicates a quick ripening process [41]. So, the result suggests that an MF treatment can reduce the production of soluble solids in tomatoes during storage and delay the activity of tissue-degrading enzymes. The difference between the MF-treated and control groups was 1.5 times. Previous studies have also shown that MF treatments, such as a 3 mT strength applied to wampees, can reduce the soluble solid content [42].

### 3.3. Effects of MF on the Tissue Degradation of Tomatoes During Storage and Enzyme Inhibition Mechanism

The cellular architecture of fruit is an intricate formation consisting of diverse polysaccharides, such as pectin and cellulose, which are fundamental in establishing the fruit’s mechanical characteristics and physiological functionalities. These constituents engage in complex interactions that significantly affect fruit texture, firmness, and overall quality throughout growth and ripening. During ripening, fruit softening is attributed mainly to the enzymatic hydrolysis of pectin within the cells [43], driven by enzymes such as PE, PG, and Cx. PE activity peaks during the early to middle ripening stages and gradually declines as the fruit matures [44]. PE facilitates the demethylation of pectin, thereby transforming it into pectic acid, which exhibits an increased vulnerability to degradation by additional enzymes such as PG. This biochemical process is essential for depolymerizing pectin, resulting in the cell wall’s loosening and fruit softening.

#### 3.3.1. Activity of the PE Enzyme

**Figure 4:**
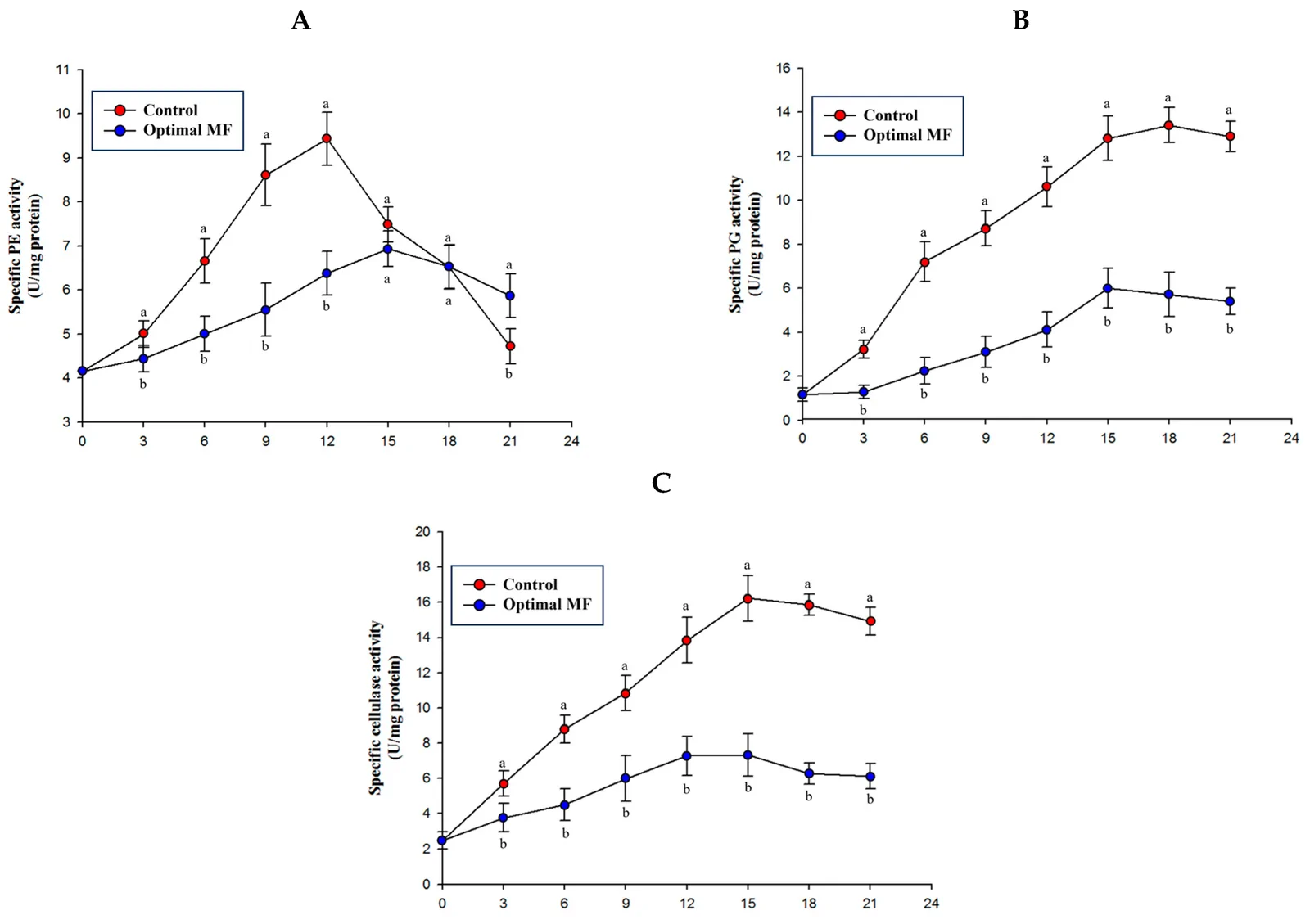
Effect of the optimal MF on the tissue deterioration enzymes (**A**) PE, (**B**) PG, and (**C**) CX of tomatoes during storage for 21 days at 25 °C (*n* = 10). The MF group was treated with an MF strength of 2 mT, frequency of 50 Hz, and treatment time of 10 min; the untreated control group was stored in an environment at 25 °C for 21 days. Letters a and b indicate statistical differences (*p* < 0.05) using unpaired *t*-test.

As shown in Figure 4A, PE activity in untreated tomatoes increased sharply with storage time, rising from 4.1 U/mg on day 0 to a peak of 9.44 U/mg on day 12. Afterward, the activity decreased to 4.21 U/mg by day 21, as the PE had hydrolyzed the pectin in the tomatoes. In contrast, the PE activity in MF-treated tomatoes remained lower than that in the control group throughout the storage period. The PE activity in the MF group peaked on day 15, with a value of 6.72 U/mg. In addition, the peak activity of the MF group was 1.5 times lower than that of the control group. These results suggest MF treatment can inhibit tomato PE activity during storage and alter the enzyme’s peak activity, delaying the pectin’s breakdown.

Xu et al. [45] demonstrated that using blue light with an intensity of 100 μmol m^2^/s inhibited PE activity in kiwifruit by approximately 12.5% compared to the untreated group during 12 days of storage (22 ± 1 °C). In comparison, the MF treatment in our study was more effective in inhibiting peak PE activity by approximately 30% relative to the untreated group. The results of this study also surpassed those of an electric field treatment in delaying the peak PE activity in tomato fruit, which was observed on the 12th day of storage [22]. The MF treatment in the present study reduced PE activity by 25% compared to an electric field treatment (250 kV/m for 30 min) on the 12th day of storage [22]. In addition, MF treatment is also better at inhibiting PE activity than chemical treatments, such as CaCl2, which can only inhibit the PE activity of litchi fruit by about 10% during storage [46].

Sun et al. [47] demonstrated that an MF (3 mT, 0.5 h) induced structural changes in PE, notably a reduction in β-sheet content accompanied by an increase in random coil and β-turn structures. In addition, MF treatment increases the hydrophobicity of specific amino acid residues, such as tyrosine and tryptophan, while phenylalanine becomes less hydrophobic. These changes may reduce conformational stability and disrupt pectinase’s active site and substrate binding, decreasing enzyme activity [47]. Furthermore, the decline in enzyme activity along with these changes was evidenced by the reduction in the kinetic parameters V_max_/K_m_ of pectinase from 0.799 to 0.366 min by the MF treatment application [47]. Therefore, we hypothesize that the inhibition of PE activity in our study is also caused by the MF’s ability to induce alterations in the secondary structure and amino acid residues’ hydrophobic interaction of PE in tomatoes [47].

#### 3.3.2. Activity of the PG Enzyme

In addition to PE, PG is also considered the primary hydrolytic enzyme in softening fruits such as tomatoes [22]. During fruit softening, pectic acid, produced by the hydrolysis of pectin by PE, is further broken down into polygalacturonic acid by PG [48], facilitating the degradation process. This process leads to accelerated softening of the fruit tissue, resulting in more severe tissue breakdown and even decay.

The results showed that the PG activity of the untreated tomato group began to increase significantly on the third day of storage (Figure 4B). The activity rose sharply with storage time, increasing from 1.32 U/mg on day 0 to a peak of 13.24 U/mg on day 18. Afterward, the activity slightly decreased to 12.11 U/mg by day 21, as the PG had hydrolyzed the polygalacturonic acid in the tomatoes. In contrast, the PG activity in MF-treated tomatoes remained lower than that in the control group throughout the storage period, with the peak activity of the MF group being 2.8 times lower than that of the control group. In addition, the enzyme activity in the MF group peaked on day 15, with a value of 5.92 U/mg. The reduction in PG activity observed with the MF treatment in our study was approximately 70% lower than that achieved with an electric field treatment (250 kV/m for 30 min) on the same storage day (day 15) [22].

Furthermore, the MF treatment demonstrated a greater efficacy in inhibiting PG activity than Nano-ZnO-based active packaging. After the storage period, PG activity in the Nano-ZnO-based packaging group was reduced by only 37.5% relative to the untreated group [49]. In contrast, the MF treatment resulted in a 67% reduction in PG activity at the same time point. These findings confirm the effectiveness of an MF treatment in inhibiting PG activity, highlighting its potential as a more efficient method for delaying fruit softening.

Sun et al. [47] demonstrated that an MF treatment can alter the polarity of aromatic amino acid residues, leading to modifications in functional groups and changes in the secondary structure of pectinase, thereby influencing its activity. This alteration in enzyme activity will inhibit the degradation of cell wall pectic polysaccharides, contributing to the delay in fruit softening. Based on these findings, we speculate that the possible factor of the PG activity inhibition in the current study is caused by the structural changes in the PG enzyme by the MF treatment, which lead to disruptions in enzyme-substrate interaction. The PG inhibition caused by an MF is also reported in [42]. In their study, an MF (3 mT MF for 30 min) inhibited PG activity by about 25% in *Clausena magnesium* (Lour.) Skeels during 12 days of storage (4 °C).

Meanwhile, in the present study, the application of an MF (2 mT, 50 Hz, 10 min) successfully inhibited the PG activity of tomatoes by 70% at room temperature. Therefore, this result highlights the superiority of our proposed treatment in PG activity inhibition compared to the previous study. However, further analysis is essential to elucidate the effects of MF treatments on the secondary structure of the PG enzyme.

#### 3.3.3. Activity of the Cx Enzyme

Figure 4C shows the Cx activity in untreated tomatoes, which began to rise significantly on the third day of storage. The activity increased sharply with time, rising from 2.53 U/mg on day 0 to a peak of 16.13 U/mg on day 15. Afterward, the activity decreased to 15.03 U/mg by day 21, as the Cx had hydrolyzed the cellulose in the tomatoes. In contrast, the Cx activity in MF-treated tomatoes remained lower than that in the control group throughout the storage period. The Cx activity in the MF group peaked on day 12, with a value of 6.83 U/mg. In addition, the peak activity of the MF group was 2.5 times lower than that of the control group. This suggests that an MF treatment can inhibit Cx activity in tomatoes during storage. In our study, the MF treatment effectively suppressed Cx activity by 65% at the end of the storage period. This level of inhibition was more significant than the 47% reduction in Cx activity observed in strawberries treated with a low-voltage electric field [50]. Moreover, the MF’s inhibition of Cx activity in the current study was markedly higher than that achieved by an HVEF treatment in jujubes [51], which resulted in only a 7.69% reduction at the end of the storage period. These findings suggest that an MF treatment is highly effective in inhibiting Cx activity and delaying softening in climacteric fruits, particularly tomatoes, during storage.

Cx is another key enzyme that degrades plant tissue’s structural integrity [52]. During the ripening process of climacteric fruits, in addition to PE and PG, Cx plays a significant role in fruit softening by hydrolyzing cellulose. This breakdown of cellulose into oligosaccharides and monosaccharides contributes to the degradation of the cell wall, further facilitating the softening process [53]. Regarding MF inhibition of Cx activity, Sun et al. [47] reported that MFs can induce conformational changes in enzymes, thereby affecting their activity. For instance, alterations in the secondary structure of cellulase, including changes in β-sheet and random coil content, have been observed under MF exposure. Similar to the effects observed in pectinase, MFs may induce structural modifications in Cx that affect the enzyme’s active site and substrate binding, ultimately leading to altered enzymatic activity [47]. Moreover, Qu et al. [19] suggested that an MF application can lead to changes in enzyme kinetic parameters (K_m_ and V_max_), such as Cx, further supporting the inhibition of Cx activity. To sum up, MF treatment effectively inhibited the activity of key tissue-degrading enzymes, including PE, PG, and Cx, thereby extending the shelf life of tomatoes. This approach holds promise as a potential future method for reducing post-harvest losses and minimizing waste in climacteric fruit storage.

#### 3.3.4. Enzyme Inhibition Mechanism

Observations on inhibiting tomato aging through an MF treatment have demonstrated its efficacy in suppressing enzyme activity and delaying the peak of enzymatic activity. Building on previous findings, enzyme kinetics were employed to investigate further the mechanism by which an MF inhibits enzyme catalysis, focusing on the behavior of enzyme-substrate interactions. These interaction patterns are categorized into competitive, noncompetitive, and uncompetitive [54].

**Figure 5:**
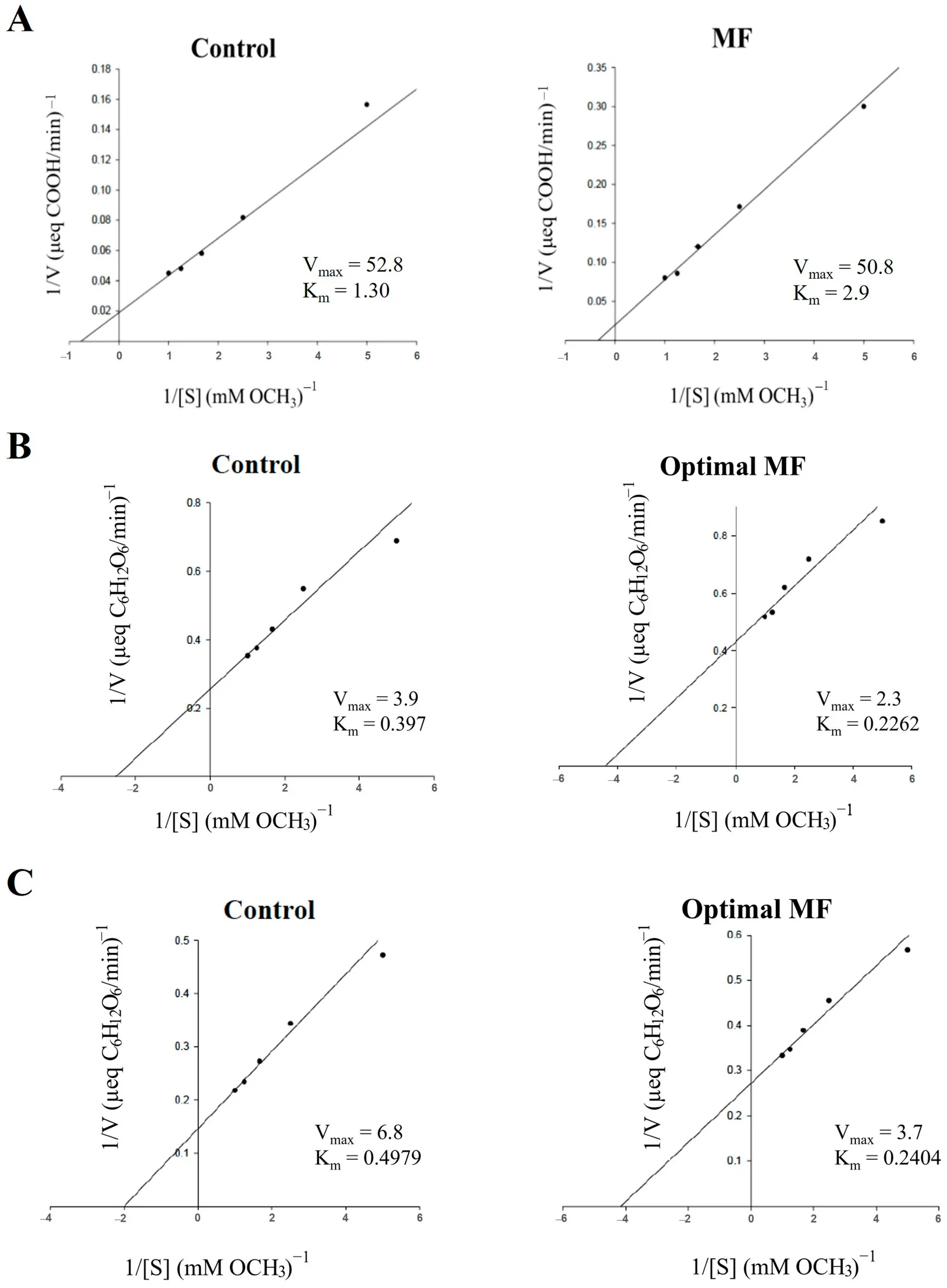
Double reciprocal plot of the activity of the tomato tissue deterioration enzymes (**A**) PE, (**B**) PG, and (**C**) Cx after optimal MF treatment (*n* = 3). V_max_ is the maximum velocity of the reaction, and K_m_ is the concentration of the substrate that permits the enzyme to achieve half V_max_.

**Figure 6:**
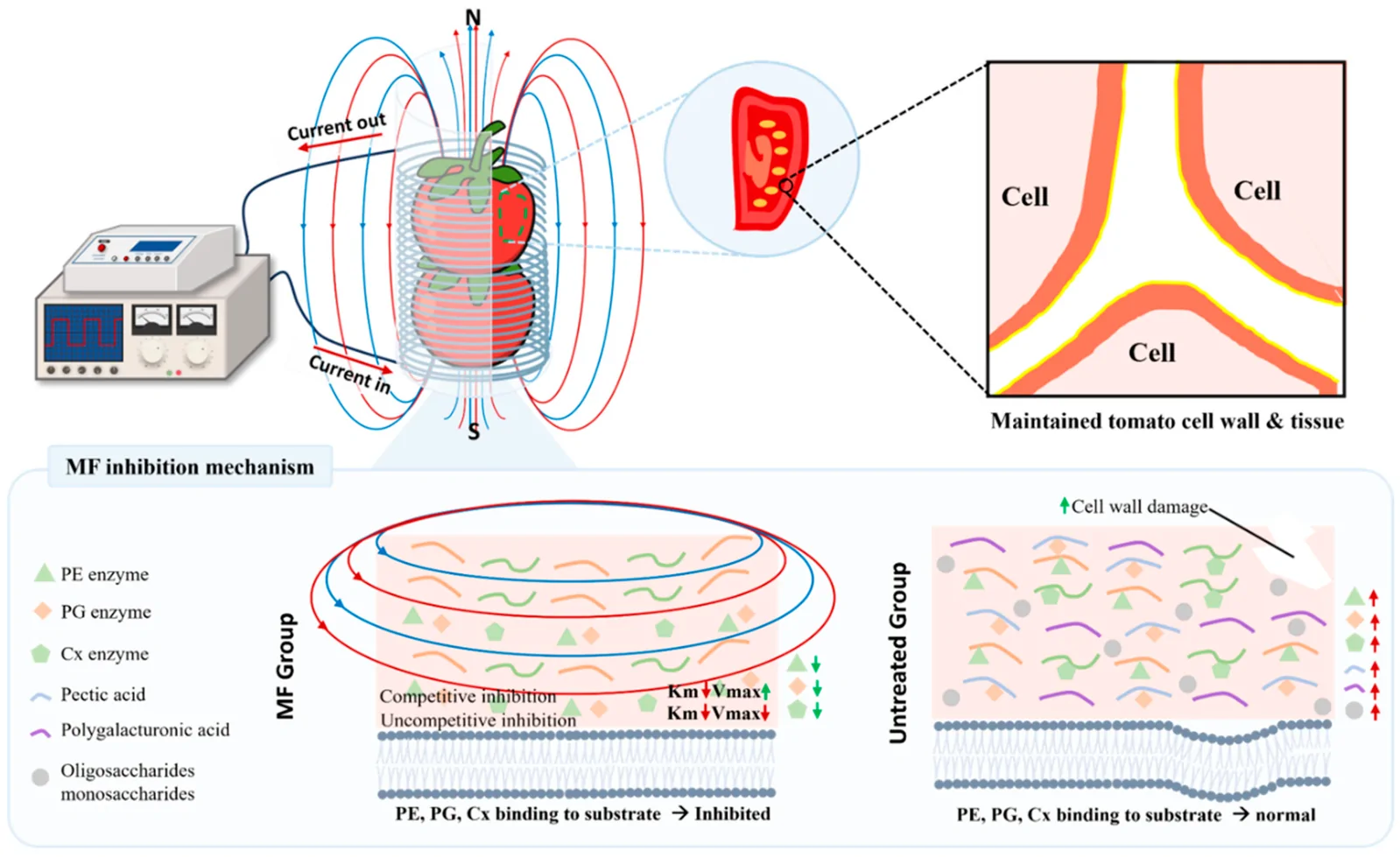
The proposed mechanism of the MF inhibits tomato tissue degradation enzymes (PE, PG, and Cx). Red and green arrows indicate decreases and increases, respectively.

As illustrated in Figure 5A, the PE kinetic parameters for the control and MF-treated samples were analyzed using titratable acidity as a critical indicator. The results revealed an increase in K_m_, from 1.3 to 2.9, alongside a modest decrease in V_max_ from 52.8 Unit/mL to 50.8 Unit/mL which indicate that the MF’s inhibition of PE activity can be categorized as a competitive inhibition type [55]. Furthermore, the decrease in the K_m_ from 0.397 to 0.226 and V_max_ from 3.9 Unit/mL to 2.3 Unit/mL of PG suggests that the MF’s inhibition of PG activity can be categorized as an uncompetitive inhibition type. Similar trends of PG (Figure 5B) are also observed in the Cx result (Figure 5C). The K_m_ declined from 0.497 to 0.24, and V_max_ decreased from 6.8 Unit/mL to 3.7 Unit/mL, indicating a similar type of uncompetitive inhibition. Since the inhibition mechanism of the MF to PG and Cx is identified as uncompetitive present study, it does not interfere with the enzyme’s active site or the substrate, and the final product output potentially remains consistent. This mechanism suggests that MF treatment can effectively preserve climacteric fruits [47]. The proposed mechanism of the MF inhibiting the tissue degradation enzymes is shown in Figure 6.

## 4. Conclusions

The MF preserved the post-harvest quality of a climacteric fruit (tomatoes). The results indicate that the optimized MF treatment (2 mT, 50 Hz, 10 min) effectively reduced weight loss in tomatoes during a 21-day storage period, delaying the rate of weight loss by approximately 1.6 times. Both external appearance and cross-sectional images confirmed that MF-treated tomatoes maintained their internal tissue structure over the storage duration. Furthermore, this treatment significantly inhibited tissue-degrading enzymes, with reductions observed in PE activity of 1.5 times, PG by 2.8 times, and Cx by 2.5 times, resulting in a prolonged shelf life compared to the untreated group. Based on a reciprocal plot analysis, it was determined that the possible inhibition mechanism of the MF on PG and Cx enzymes is uncompetitive, making it potential ideal approach for extending the shelf life of climacteric fruits while allowing natural ripening without compromising flavor. Future research analyzing potential climacteric respiration as affected by an MF (e.g., by measuring reactive oxygen species, oxygen, and ethylene) is suggested to achieve practical application. This study contributes to more efficient and sustainable post-harvest preservation strategies for climacteric fruits by advancing understanding of MF applications and mechanisms. Future up-scale MF studies are crucial for evaluating its effectiveness and readiness for actual application.

## Figures and Tables

**Table 1:** Orthogonal array design (OAD) and analysis of weight loss in tomatoes.

Sample Runs	Factors			Result
	Strength (mT)	Frequency (Hz)	Time (min)	Weight Loss (%)
1	1	0	10	6.37 ± 0.11
2	1	50	20	5.13 ± 0.09
3	1	100	30	5.56 ± 0.14
4	2	0	20	4.85 ± 0.13
5	2	50	30	5.33 ± 0.15
6	2	100	10	5.05 ± 0.16
7	3	0	30	6.42 ± 0.17
8	3	50	10	4.52 ± 0.12
9	3	100	20	6.41 ± 0.11
k1	5.636	5.934	5.290	
k2	5.110	4.956	5.471	
k3	5.780	5.636	5.764	
R (Δ)	0.670	0.979	0.474	
Rank	2	1	3	

Tomatoes were treated within the MF group with 9 experimental runs (1, 2, 3, 4, 5, 6, 7, 8, 9). The data are expressed as the mean ± SD (*n* = 10). k1, k2, and k3 are the means of the weight loss percentage derived from their respective factor level. R (delta) subtracted the highest and lowest k1, k2, and k3 in each factor, indicating the factors’ contribution to the treatment outcome.

**Table 2:** Variance analysis of the orthogonal array design (OAD) of the optimal MF treatment variables in relation to weight loss in tomatoes.

Source	DF	SS	MS	F-Value	*p*-Value	C (%)
Strength (mT)	2	2.238	1.1190	2.33	0.119	24.91
Frequency (Hz)	2	4.530	2.2649	5.87	0.008	50.42
Time (min)	2	1.032	0.5159	0.97	0.393	11.48
Error	2	1.8335	1.8335	0.9167		
Total	8	8.98335				

DF: degrees of freedom; SS: sum of the square; MS: mean of the square; C (%): contribution percentage.

## Data Availability

The original contributions presented in this study are included in the article; further inquiries can be directed to the corresponding authors.
