# Correction: Chang et al. Optimizing Non-Thermal Magnetic Field to Minimize Weight Loss and Tissue Degradation: Identifying Possible Enzyme Inhibition Mechanisms. *Foods* 2025, *14*, 166

**DOI:** 10.3390/foods15152699

**Published:** 2026-07-31

**Authors:** Chao-Kai Chang, Prakoso Adi, Rizka Mulyani, Chun-Fu Lin, Ratna Sari Listyaningrum, Shella Permatasari Santoso, Mohsen Gavahian, Chang-Wei Hsieh

**Affiliations:** 1 Department of Food Science and Biotechnology, National Chung Hsing University, Taichung City 402202, Taiwan; 2 International Doctoral Program in Agriculture, National Chung Hsing University, Taichung City 402202, Taiwan; prakoso.adi@staff.uns.ac.id (P.A.); rizka.mulyani@staff.uns.ac.id (R.M.);; 3 Department of Agricultural Product Technology, Sebelas Maret University, Surakarta City 57126, Indonesia; 4 Department of Medicinal Botanicals and Health Applications, Da-Yeh University, Chang-Hua 515006, Taiwan; 5 Department of Food Technology, Muhammadiyah University of Bandung, Bandung City 40614, Indonesia; 6 Department of Chemical Engineering, Widya Mandala Surabaya Catholic University, Surabaya 60114, Indonesia; shella@ukwms.ac.id; 7 Department of Chemical Engineering, National Taiwan University of Science and Techology, Taipei 106335, Taiwan; 8 Department of Food Science, National Pingtung University of Science and Technology, Pingtung 912301, Taiwan; 9 Department of Food Science, National Ilan University, Yilan City 260007, Taiwan; 10 Department of Medical Research, China Medical University Hospital, Taichung City 404327, Taiwan

## 1. Text Correction

There were some errors in the original publication [1], that have been hereby corrected, as detailed below.

A correction has been made to Section 2.7. Statistical Analysis, Paragraph 1, to specify the number of replicates:

The first sentence “Data were expressed as the mean ± standard deviation (SD) from ten replications.” has been removed and a new description sentence has been added to the end of the paragraph: “Ten replicates were used to calculate the mean ± standard deviation (SD) for the preliminary experiments, the selection of optimal MF conditions, the assessment of quality attributes during storage, including weight loss, soluble solids, and titratable acidity, and the analysis of tissue-degrading enzyme activities; whereas three replicates were used to calculate the mean ± SD for the analysis of the enzyme inhibition.”

Since the Km values for the control and MF treatment in Figure 5A of the original published article were inadvertently interchanged, the reported Km values and the corresponding interpretation of the enzyme inhibition mechanism require correction. A correction has been made to Section 3.3.4. Enzyme Inhibition Mechanism, Paragraph 2 and Section 4. Conclusions for further clarifications.

The second to the sixth sentences in Section 3.3.4., Paragraph 2, has been updated to: “The results revealed an increase in K_m_, from 1.3 to 2.9, alongside a modest decrease in V_max_ from 52.8 Unit/mL to 50.8 Unit/mL which indicate that the MF’s inhibition of PE activity can be categorized as a competitive inhibition type [55]. Furthermore, the decrease in the K_m_ from 0.397 to 0.226 and V_max_ from 3.9 Unit/mL to 2.3 Unit/mL of PG suggests that the MF’s inhibition of PG activity can be categorized as an uncompetitive inhibition type. Similar trends of PG (Figure 5B) are also observed in the Cx result (Figure 5C). The K_m_ declined from 0.497 to 0.24, and V_max_ decreased from 6.8 Unit/mL to 3.7 Unit/mL, indicating a similar type of uncompetitive inhibition. Since the inhibition mechanism of the MF to PG and Cx is identified as uncompetitive present study, it does not interfere with the enzyme’s active site or the substrate, and the final product output potentially remains consistent.”

The fifth sentence in Section 4 has been updated to “Based on a reciprocal plot analysis, it was determined that the possible inhibition mechanism of the MF on PG and Cx enzymes is uncompetitive, making it potential ideal approach for extending the shelf life of climacteric fruits while allowing natural ripening without compromising flavor.”

## 2. Figures Corrections

In the original publication [1], Figures 5A and 6 need the following corrections.

In Figure 5A of the original published article, the K_m_ values for the control and MF treatments were inadvertently interchanged. The correct K_m_ values are 1.30 for the control and 2.90 for MF treatment. Also, the figure footer has also been updated to include the number of replicates. The corrected Figure 5 and its caption appear below.

In addition, since the K_m_ values for the control and MF treatment in Figure 5A of the original published article were inadvertently interchanged, the proposed mechanism of the MF inhibiting the tissue degradation enzymes shown in Figure 6 should be updated accordingly for further clarifications. The corrected Figure 6 appears below.

The authors state that the scientific conclusions are unaffected. This correction was approved by the Academic Editor. The original publication has also been updated.

## Figures and Tables

**Figure 5:**
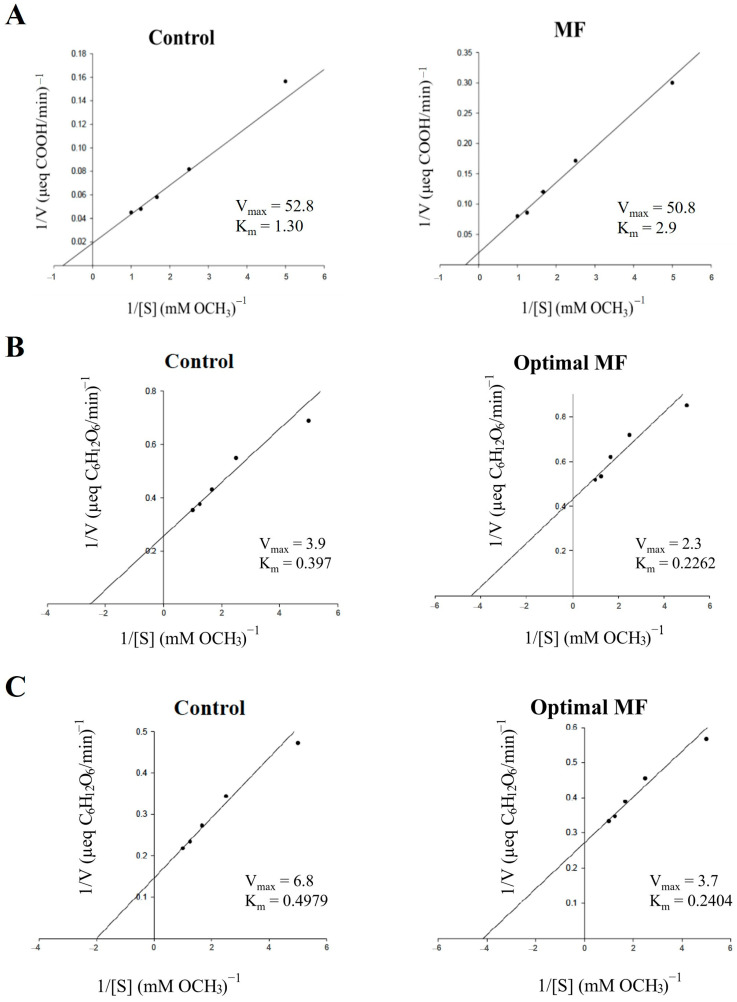
Double reciprocal plot of the activity of the tomato tissue deterioration enzymes (**A**) PE, (**B**) PG, and (**C**) Cx after optimal MF treatment (*n* = 3). V_max_ is the maximum velocity of the reaction, and K_m_ is the concentration of the substrate that permits the enzyme to achieve half V_max_.

**Figure 6:**
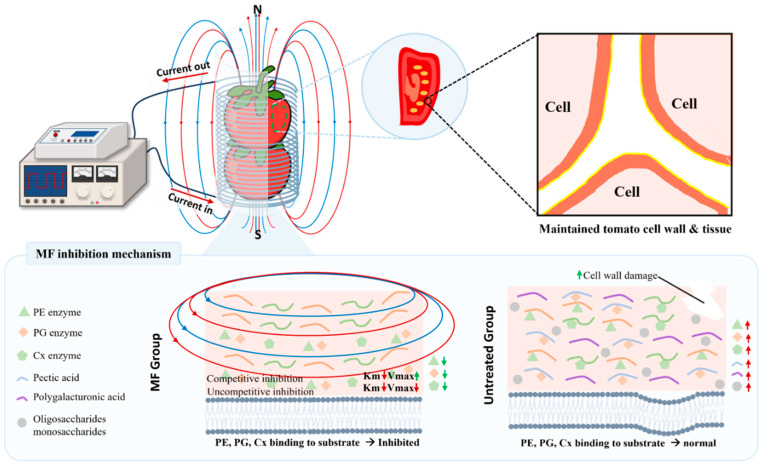
The proposed mechanism of the MF inhibits tomato tissue degradation enzymes (PE, PG, and Cx). Red and green arrows indicate decreases and increases, respectively.
